# Validation of an analytical method based on the high-resolution continuum source flame atomic absorption spectrometry for the fast-sequential determination of several hazardous/priority hazardous metals in soil

**DOI:** 10.1186/1752-153X-7-43

**Published:** 2013-03-01

**Authors:** Tiberiu Frentiu, Michaela Ponta, Raluca Hategan

**Affiliations:** 1 Faculty of Chemistry and Chemical Engineering, Babes-Bolyai University, 11 Arany Janos, 400028 Cluj-Napoca, Romania; 2 Regional Environmental Protection Agency, 99 Dorobantilor, 400609 Cluj-Napoca, Romania

**Keywords:** High-resolution continuum source flame atomic absorption spectrometry, Inductively coupled plasma optical emission spectrometry, Bland and Altman statistical method, Soil analysis, Hazardous/priority hazardous metal

## Abstract

**Background:**

The aim of this paper was the validation of a new analytical method based on the high-resolution continuum source flame atomic absorption spectrometry for the fast-sequential determination of several hazardous/priority hazardous metals (Ag, Cd, Co, Cr, Cu, Ni, Pb and Zn) in soil after microwave assisted digestion in aqua regia. Determinations were performed on the ContrAA 300 (Analytik Jena) air-acetylene flame spectrometer equipped with xenon short-arc lamp as a continuum radiation source for all elements, double monochromator consisting of a prism pre-monocromator and an echelle grating monochromator, and charge coupled device as detector. For validation a method-performance study was conducted involving the establishment of the analytical performance of the new method (limits of detection and quantification, precision and accuracy). Moreover, the Bland and Altman statistical method was used in analyzing the agreement between the proposed assay and inductively coupled plasma optical emission spectrometry as standardized method for the multielemental determination in soil.

**Results:**

The limits of detection in soil sample (3σ criterion) in the high-resolution continuum source flame atomic absorption spectrometry method were (mg/kg): 0.18 (Ag), 0.14 (Cd), 0.36 (Co), 0.25 (Cr), 0.09 (Cu), 1.0 (Ni), 1.4 (Pb) and 0.18 (Zn), close to those in inductively coupled plasma optical emission spectrometry: 0.12 (Ag), 0.05 (Cd), 0.15 (Co), 1.4 (Cr), 0.15 (Cu), 2.5 (Ni), 2.5 (Pb) and 0.04 (Zn). Accuracy was checked by analyzing 4 certified reference materials and a good agreement for 95% confidence interval was found in both methods, with recoveries in the range of 94–106% in atomic absorption and 97–103% in optical emission. Repeatability found by analyzing real soil samples was in the range 1.6–5.2% in atomic absorption, similar with that of 1.9–6.1% in optical emission spectrometry. The Bland and Altman method showed no statistical significant difference between the two spectrometric methods for 95% confidence interval.

**Conclusions:**

High-resolution continuum source flame atomic absorption spectrometry can be successfully used for the rapid, multielemental determination of hazardous/priority hazardous metals in soil with similar analytical performances to those in inductively coupled plasma optical emission spectrometry.

## Background

Twenty five years ago low-resolution line source atomic absorption spectrometry (LR-LS AAS), although in full maturity and providing high sensitivity and selectivity through the element-specific line radiation source, had limited versatility and low speed of analysis being known as a single-element technique. To face the challenges of the inductively coupled plasma optical emission spectrometry (ICP-OES), which imposed itself as a fast multielemental technique due to the charged-coupled device (CCD) technology, the need to increase the versatility of AAS with respect to line selection, background correction and higher analysis speed became imperative. Thus, in the mid-1990s a research group led by Becker-Ross from the Institute for Analytical Sciences, Berlin, Germany, designed and built the first instrument for high-resolution continuous source atomic absorption spectrometry (HR-CS AAS), in which all components were optimized for the novel approach [1-3]. The new concept instrument has been for several years commercially available for both flame (HR-CS FAAS) and graphite furnace (HR-CS GFAAS) atomization. The relevant advantages of HR-CS AAS over LR-LS AAS are [4]: (i) use of just one source for all elements; (ii) visualization of the environment of the analytical line permitting superior background correction by simultaneous measurement of atomic and background absorption; (iii) fast-sequential multielemental determination using atomic lines or molecular bands (changing lines and parameters optimization only takes a few seconds); (iv) correction of the fine-structured background using a reference spectrum. The extremely well acceptance of HR-CS AAS instrument after its introduction on the market together with the versatility of the analytical applications was reflected in several reviews [5-11]. Both approaches of the technique, using either flame or electrothermal atomization, were used for multielemental determination in various complex matrices. Thus, HR-CS GFAAS was mentioned for elemental determination in food after acidic digestion or extraction in tetramethylammonium hydroxide [12-14], airborne particulates [15-18], biological (beans and grain) samples using direct solid sampling [19-21], fertilizer by slurry sampling [22], crude oil [23-26], biodiesel [27] and in whole blood directly in diluted/undiluted samples [28].

At the same time, HR-CS FAAS was used for multielemental determination in vegetable oil as microemulsion [29], plant tissues after mineralization [30,31], dairy products by slurry sampling [32], pewter alloys after digestion in HCl solution [33], lubricating oil after acid digestion, oil-in water emulsification and dilution in kerosene [34-36], and soil extracts [37].

The introduction of the continuum source and high-resolution monochromator has opened the door to monitor non-metals (P, S, F, Br, N, etc.) with sensitive lines in the far UV, not accessible for LS-AAS. Thus, the molecular absorption spectrometry (HR-CS MAS) can overcome this barrier by measuring the absorption of a line within the band spectra of a diatomic molecular radical (PO, CS, SH, AlF, SrF, GaF, AlBr, CaBr, NO, etc.) stable in flame or graphite furnace at the atomizer temperature [9,21,38-49].

This paper reports on the validation of an analytical method for the fast-sequential determination of several hazardous/priority hazardous metals (Ag, Cd, Cr, Co, Cu, Ni, Pb and Zn) in soil based on the HR-CS FAAS. The soil samples were subjected to microwave assisted digestion in aqua regia according to ISO 11466 – 1999. Validation was conducted according to the method-performance study to estimate the performance characteristics of the new method (limit of detection, limit of quantification, precision and accuracy [50,51]. Furthermore, the Bland and Altman statistical method was used in analyzing the agreement between the proposed assay and inductively coupled plasma optical emission spectrometry as standardized method for the multielemental determination in soil [52].

The paper is important for the analytical practice because currently the standardized methods for soil analysis by atomic absorption spectrometry are LR-LS FAAS and GFAAS [53]. On the other hand, ISO/IEC 17025–2005 and the Programme of Association of Official Analytical Chemistry demand the fit-for-purpose validation of the new techniques/assays before use.

## Results and discussion

### Limits of detection and quantification

The limits of detection and quantification found in HR-CS FAAS and ICP-OES according to the 3σ criterion are given in Table 1. The limit of detection (3σ_b_ /m) was computed considering the slope of the calibration curve (m) and the standard deviation of the background (σ_b_) from 10 successive measurements of the signal corresponding to blank [50,51,54,55]. The blank was prepared in the same way as the soil samples using identical volumes of reagents. The limit of quantification corresponding to the lowest concentration being determined with a specified relative standard deviation, commonly 10%, was considered as 3 times the limit of detection. Based on the protocol of sample preparation, the limits of detection and quantification were expressed in dry mass (mg/kg). The calibration curves in HR-CS FAAS drawn on the range 0 – 1 μg/ml element had correlation coefficients of 0.9950 - 0.9999, close to those found in ICP-OES. The limits of quantification found in the analytical method based on HR-CS FAAS allow the determination of elements in soil at concentration levels below the limits of applicability given for LR-LS FAAS [53]. Data in Table 1 reveals better detection limits for Cr, Cu, Ni and Pb by HR-CS FAAS than by ICP-OES. A comparison of the detection limits found by us with those given in [52] for ICP-OES is not relevant, since the values are strongly depended on equipment and laboratory conditions. However, each laboratory should determine its specific LODs within a validation study.

**Table 1:** Limits of detection (3σ criterion) and quantification (mg/kg) in soil

	**HR-CS-FAAS**			**ICP-OES**			**Limit of applicability of LR-LS-FAAS**^**c**^
	**λ (nm)**	**LOD**^**a**^	**LOQ**^**b**^	**λ (nm)**	**LOD**^**a**^	**LOQ**^**b**^	
Ag	328.068	0.18	0.54	328.068	0.12	0.36	-
Cd	228.802	0.14	0.42	214.438	0.05	0.15	>2
Co	240.206	0.36	1.08	228.615	0.15	0.45	>12
Cr	357.869	0.25	0.75	267.716	1.4	4.2	>12
Cu	324.754	0.09	0.27	324.754	0.15	0.45	>5
Ni	232.003	1.0	3.0	305.082	2.5	7.5	>12
Pb	217.000	1.4	4.2	220.351	2.5	7.5	>15
Zn	213.856	0.18	0.54	213.856	0.04	0.12	>2

^a^ – LOD calculated for an amount of 1.000 g soil mineralized and diluted to 100 ml solution.

^b^ – LOQ considered three times LOD.

^c^ – according to [53].

### Accuracy of the multielemental determinations

Accuracy of the analytical method based on HR-CS FAAS was assessed by analyzing 4 certified reference samples. The results in comparison with those found in ICP-OES are displayed in Table 2. The average recovery and pooled standard deviation of 95% confidence interval were computed based on the results obtained for each CRM. According to data in Table 2, the analytical method based on HR-CS FAAS provided good accuracy with recoveries in the range 94–106%, similar to the values of 97–103% found in ICP-OES. The good recovery of certified value in both methods indicated the absence of interferences. Moreover, the method of correction the continuous background and NO molecular absorption in HR-CS FAAS proved to be efficient in the case of the complex matrix of soil.

**Table 2:** Found values (m = 5 independent samples) in the analysis of CRMs compared to certified values (mg/kg)

	**BCR 025-050**^**a**^			**LGC 6135**^**a**^			**LGC 6141**^**a**^			**NCSDC**			**Recovery**^**b**^**(%)**	
	**Found**		**Certified**	**Found**		**Certified**	**Found**		**Certified**	**Found**		**Certified**	**ICP-OES**	**HR-CS AAS**
	**ICP-OES**	**HR-CS AAS**		**ICP-OES**	**HR-CS AAS**		**ICP-OES**	**HR-CS AAS**		**ICP-OES**	**HR-CS AAS**			
Cd	340 ± 45.0	383 ± 76.0	369 ± 46.3	-		-	-	-	-	2.50 ± 0.5	2.40 ± 0.2	2.45 ± 0.3	97 ± 17	101 ± 15
Co	4.00 ± 0.80	3.98 ± 0.80	4.07 ± 0.93	19 ± 3	16 ± 4	20 ± 4	-	-	-	17.1 ± 1.5	17.0 ± 2.0	16.5 ± 1.8	99 ± 16	94 ± 20
Cr	448 ± 41.0	403 ± 34.0	441 ± 50.1	349 ± 12	344 ± 30	336 ± 28	120 ± 20	115 ± 20	130 ± 31	88 ± 5	105 ± 6	90 ± 8	99 ± 10	100 ± 11
Cu	8.35 ± 1.68	9.62 ± 1.60	7.76 ± 1.68	100 ± 5	102 ± 11	105 ± 5	52.2 ± 0.8	52.3 ± 7.5	51.1 ± 13	54 ± 5	51 ± 2	53 ± 6	102 ± 14	105 ± 12
Ni	12.0 ± 3.25	12.9 ± 1.80	12.2 ± 3.54	240 ± 16	275 ± 27	227 ± 13	50 ± 5	50 ± 5	49 ± 13	34 ± 6	30 ± 3	32	103 ± 17	106 ± 11
Pb	1448 ± 108	1454 ± 188	1447 ± 203	400 ± 21	395 ± 10	391 ± 16	75.1 ± 12	74.1 ± 10	75.8 ± 16	78 ± 5	75 ± 5	79 ± 12	100 ± 10	99 ± 10
Zn	55.0 ± 8.14	60.6 ± 11.05	51.8 ± 8.29	316 ± 18	339 ± 26	316 ± 41	167 ± 30	168 ± 30	169 ± 39	248 ± 38	245 ± 24	251	101 ± 14	105 ± 14

^a^ – average ± U (U – expanded uncertainty for 95% confidence level).

^b^ – average recovery for 95% confidence level.

### Precision in HR-CS FAAS and ICP-OES

The metal contents and standard deviations found in soil samples by HR-CS FAAS and ICP-OES are presented in Additional file 1. The pooled relative standard deviations in HR-CS FAAS were: 2.5% (Ag), 4.6% (Cd), 3.2% (Cr), 4.3% (Cu), 5.2% (Co), 3.2% (Ni), 2.2% (Pb), 1.6% (Zn), similar to those in ICP-OES: 4.7% (Ag), 3.2% (Cd), 2.3% (Cr), 6.1% (Cu), 4.4% (Co), 4.9% (Ni), 3.7% (Pb) and 1.9% (Zn). The values comply with the informative ones given in [52].

### Statistical validation using the Bland and Altman method

**Figure 1:**
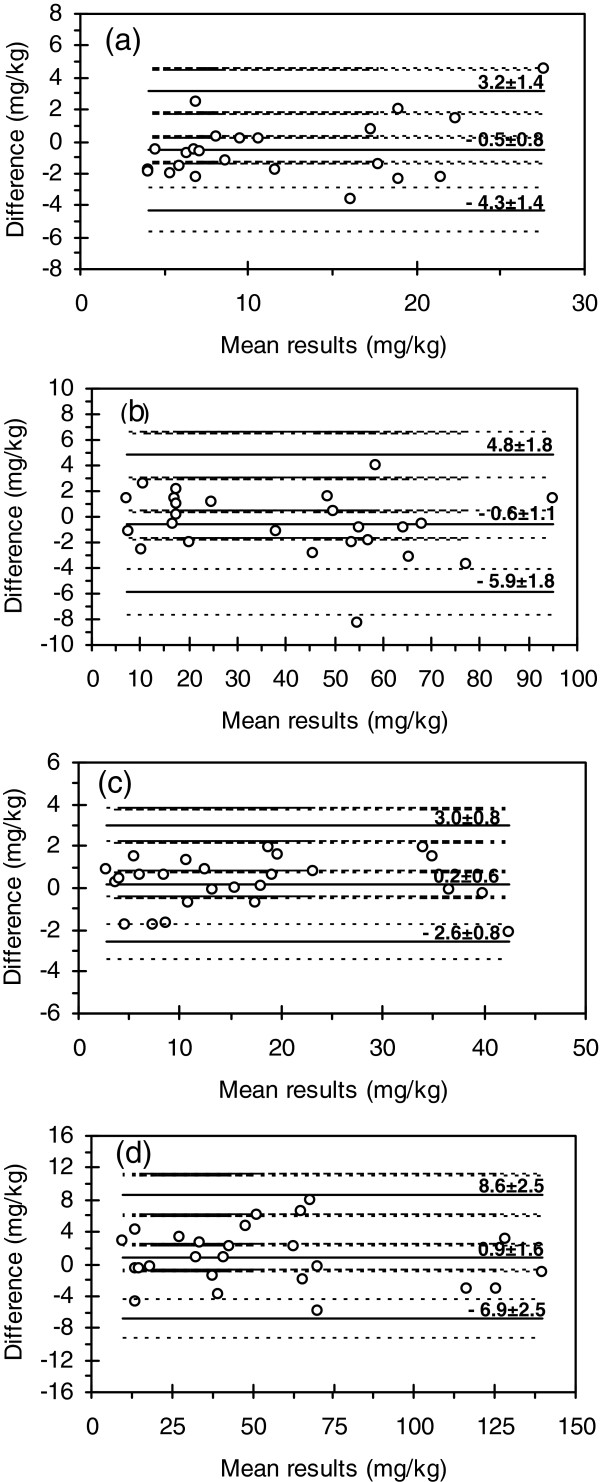
Bland and Altman plots: (a) Ag (n=25); (b) Cd (n=25); (c) Co (n=25); (d) Cr (n=25). n– sample size.

The Bland and Altman plots are displayed in Figure 1 and Additional file 2, while the descriptive statistics for 95% confidence interval (CI) in Table 3.

**Table 3:** Descriptive statistics for the Bland and Altman test (95% confidence level, m = 3 independent samples)

**Element**	**Within-method standard deviation (mg/kg)**		**Between-method standard deviation (s**_**B**_**) (mg/kg)**	**Confidence interval of bias**$\bar{\Delta }\pm t\frac{s_{B}}{\sqrt{n}}$**(mg/kg)**	**Confidence intervals for limits of agreement**$\bar{\Delta }\pm 1.96s_{B}\pm t\cdot s_{\mathrm{LL},\mathrm{UL}}$**(mg/kg)**
	ICP-OES	HR-CS-AAS			
Ag (n = 25)	0.4	0.3	1.8	-0.5 ± 0.8	-4.1 ± 1.3
					3.1 ± 1.3
Cd (n = 25)	1.0	0.8	2.7	-0.6 ± 1.1	-5.9 ± 1.8
					4.8 ± 1.8
Co (n = 25)	0.7	0.7	1.4	0.2 ± 0.6	-2.6 ± 0.8
					3.0 ± 0.8
Cr (n = 25)	1.3	1.4	3.9	0.9 ± 1.6	-6.9 ± 2.5
					8.6 ± 2.5
Cu (n = 34) (<1000)^a^	30.1	18.7	33.4	4.8 ± 11.7	-60.8 ± 14.0
					70.3 ± 14.0
Cu (n = 16) (1000–22000)^a^	260	246	390	-57 ± 208	-821 ± 258
					707 ± 258
Ni (n = 25)	0.9	1.9	2.5	-0.4 ± 1.0	-5.3 ± 1.3
					4.5 ± 1.3
Pb (n = 28) (<1000)^a^	29.6	22.9	42.5	4.9 ± 16.5	78.3 ± 20.8
					88.2 ± 20.8
Pb (n = 14) (1000–10000)^a^	174	56	214	-36 ± 123	-455 ± 162
					383 ± 162
Pb (n = 13) (10000–60000)^a^	712	494	1477	-243 ± 893	-3138 ± 1343
					2652 ± 1343
Zn (n = 30) (< 1000)^a^	7.5	5.2	13.2	-3.3 ± 4.9	-29.1 ± 6.9
					22.6 ± 6.9
Zn (n = 20) (> 1000)^a^	122	90	169	13 ± 79	-319 ± 100
					345 ± 100

^a^ – concentration range in mg/kg; n – sample size.

The contents of 3 out of the 8 elements under study (Cu, Pb and Zn) covered a wide range of concentration with individual values grouped on specific sub-domains. Therefore, in the case of Cu, the samples were split in two groups, with contents below 1000 mg/kg (34 samples) and in the range 1000 – 22000 mg/kg (16 samples). The distribution of Pb contents imposed the grouping in three sub-groups: below 1000 mg/kg (28 samples), in the range 1000 – 10000 mg/kg (14 samples) and 10000 – 60000 mg/kg (13 samples), respectively. Sample grouping in two sub-domains was also used in respect with Zn, namely below 1000 mg/kg (30 samples) and over 1000 mg/kg (20 samples). The inspection of narrower ranges of concentration provided a more accurate assessment of results found in HR-CS FAAS and ICP-OES. Plots in Figure 1, Additional file 2 and data in Table 3 demonstrate a good agreement between methods for 95% CI.

The positive bias for Co and Cr (Figure 1c,d), Cu and Pb < 1000 mg/kg and Zn > 1000 mg/kg (see Additional file 2e,h,l) and the negative one for Ag, Cd (Figure 1a,b), Cu > 1000 mg/kg, Ni, Pb in the range 1000 – 60000 mg/kg and Zn < 1000 mg/kg (see Additional file 2f,g,i,j,k) towards the ICP-OES method was low in relation to the concentrations of the elements. In all these cases the 95% CI of the bias included the zero value, so that errors were random and the null hypothesis was retained. With respect to Co and Cr (Figure 1c,d), Cu (whole concentration range), Ni, Pb (whole concentration range) and Zn (1000 – 11000 mg/kg) (see Additional file 2e,f,g,h,i,j,l), all results fall within the limits of agreement of the differences between methods. In the case of Ag (Figure 1a) one result out of 25 was above the upper limit of agreement, while for Zn 2 out of the 20 values > 1000 mg/kg (see Additional file 2,l) surpassed the upper limit and 1 the lower one. However, in all cases the values remained within the 95% CI of the limits. In short, the differences between results for Zn by HR-CS FAAS and ICP-OES were random and the null hypothesis was retained over the whole concentration range.

Although in the case of Cd (Figure 1b), one of the 25 results was above the upper limit of agreement and outside its 95% CI, the agreement between HR-CS FAAS and ICP-OES could be considered good.

## Conclusions

An analytical method based on the HR-CS FAAS was validated for the fast-sequential determination of eight hazardous/priority hazardous elements (Ag, Cd, Co, Cr, Cu, Ni, Pb and Zn) in soil samples digested in aqua regia. The study revealed similar analytical performances to those in ICP-OES, the standardized method for the multielemental determination in soil and considered as reference in this study. The statistical analysis using the Bland and Altman test demonstrated a good agreement between the two methods over a wide concentration range for 95% CI. In these circumstances it is obvious that the new instrumental concept based on HR-CS FAAS recently commercially available can be successfully used for the determination of hazardous/priority hazardous metals in soil with performances similar to those reported in ICP-OES. The main contribution of the paper was broadening the applicability of HR-CS FAAS for the determination of hazardous/priority hazardous metals in soil, very attractive by its fast, multielemental capability and easy to handle with respect to optimization and overcoming spectral interferences. In the same time, the use of the Bland and Altman test allowed the comparison of results over a large concentration range.

## Experimental

### Reagents, standards and CRMs

Nitric acid, 65% and hydrochloric acid, 37% of analytical grade (p.a.) (Merck, Darmstadt, Germany) were used to mineralize the soil samples. Argon 5.0 quality and acetylene 4.5 quality (Linde Gas SRL Cluj-Napoca, Romania) were used as working gases to generate ICP and flame, respectively. The accuracy of the HR-CS FAAS method was checked by analyzing three certified reference samples of soil containing priority contaminants, CRM 025–050, LGC 6135, LGC 6141, and one river sediment, NCSDC 78301 (LGC Promochem GmbH, Wessel, Germany). High purity water (18 MΩ cm^-1^) from a Milli Q system (Millipore, Bedford MA, USA) was used throughout.

The ICP multielement standard solution IV 1000 μg/ml (Merck, Darmstadt, Germany) was used to prepare multielement working standards in the range 0 – 1 μg/ml by dilution with 2% HNO_3_. A blank calibration solution of 2% HNO_3_ was used in recording the reference spectrum for correction NO molecular absorption on Zn 213.856 nm line.

## Instrumentation

### Flame atomic absorption spectrometer

Measurements in FAAS were carried out using the ContrAA 300 high-resolution continuum source flame atomic absorption spectrometer from Analytik Jena AG (Jena, Germany), currently the only manufacturer of such instrumentation. The spectrometer is equipped with a xenon short-arc lamp XBO 301 (GLE, Berlin, Germany) with a nominal power of 300 W operating in a hot-spot mode suitable for all elements, a double monochromator consisting of a prism pre-monochromator and an echelle grating monochromator (resolution 1.5 pm per pixel in the far UV and 3 pm at 400 nm) and CCD array with 588 pixels. The detector allows visualization of the environment of the analytical line using 200 pixels. Five pixels in the centre of the window were used to measure atomic absorption, while the others for correction purposes. The concept of ContrAA 300 provides the fast-sequential multielement determination as well as an efficient background correction by simultaneous measurement of atomic and continuous background absorption. Additionally, the interference of fine-structured background of NO molecule on Zn 213.856 nm line was eliminated based on the reference spectrum registered for a 2% HNO_3_ solution and the least-squares background correction (LSBC). The primary lines of the elements were selected for the determination due to their highest sensitivity. The working conditions recommended by the manufacturer with the HR-CS ContrAA 300 spectrometer are summarized in Table 4.

**Table 4:** Working conditions for measurements by HR-CS FAAS ContrAA 300 in air-acetylene flame

**Analyte**	**Wavelength (nm)**	**Acetylene (l/min)**	**Air (l/min)**	**Burner height (mm)**
Ag	328.068	50	500	6
Cd	228.802	50	500	6
Co	240.206	50	500	6
Cr	357.869	100	400	8
Cu	324.754	50	470	6
Ni	232.003	55	470	6
Pb	217.000	65	470	6
Zn	213.856	50	470	6

### Inductively coupled plasma optical emission spectrometer

A SPECTRO CIROS^CCD^ ICP optical emission simultaneous spectrometer with axial plasma viewing (Spectro Analytical Instruments, Kleve, Germany) was used to obtain the comparative values for the Bland and Altman test. The SPECTRO CIROS features a Paschen-Runge spectrometer mount, consisting of a double-grating optical system with 22 CCD detectors. Details about the operating conditions of the spectrometer and the working wavelengths are given in Table 5. As for HR-CS FAAS the most sensitive wavelengths were selected for emission measurements.

**Table 5:** **Operating conditions and analytical lines for the SPECTRO CIROS**^**CCD**^**ICP optical emission spectrometer**

**Component**	**Characteristic**
Generator	Free-running 27.12 MHz operated at 1400 W
Plasma torch	Axial viewing, torch positioning (mm) X = - 3.9, Y = + 3.6, Z = + 2.6.
	Argon flow rates:
	Outer gas 12 l/min
	Intermediate gas 0.6 l/min
	Nebulizer gas 1 l/min
Sample introduction system	Four channels peristaltic pump, K2 cross-flow nebulizer, double pass Scott type spray chamber, 2 ml/min sample uptake rate
Polychromator	160 – 600 nm double grating Paschen-Runge mounting, chamber filled with Ar
Detector	22 CCDs
Data processing	Background correction: linear and square two points models, best SNR strategy, integration time 48 s and three successive measurements for each sample.
Analytical wavelengths	Ag 328.068 nm; Cd 214.438 nm; Co 228.615 nm; Cr 267.716 nm; Cu 324.754 nm; Ni 305.082 nm; Pb 220.351 nm; Zn 213.856

### Instrumentation used in sample preparation

A mortar grinder Retsch RM 1000 and a sieve shaker Retsch 200 (Hann, Germany), a Memmert UFE 500 oven (Schwabach, Germany), a high-pressure (100 atm) Berghof MW-S3+ system with control temperature until 210°C (Eningen, Germany) and a Sartorius vacuum filter equipment (Goettingen, Germany) were used for sample preparation and mineralization.

## Methods

### Soil sample preparation

Mineralization of soil samples was performed according to ISO 11466-1999. Samples were dried for 4 h at 105 ± 5°C, then crushed and sieved through a 2-mm sieve to remove stones. The fraction < 2 mm was ground and sieved again through a 250-μm sieve. Amounts of 1.0000 g soil sample prepared in this way were subjected to mineralization with 10 ml aqua regia in closed PTFE containers of the microwave digester according to the protocol given in Table 6. The digest was transferred to a 100 ml volumetric flask and diluted with 2% HNO_3_ to mark. After filtration, the solution was kept in polyethylene flask until analysis. Elements were determined by HR-CS FAAS and ICP-OES in 3 independent samples and 3 successive measurements on each sample. Dilutions were carried out so that sample concentration fall within the calibration curve.

**Table 6:** Operating conditions for the microwave digestion system

	**Step**			
	**1**	**2**	**3**	**4**
Temperature (°C)	180	100	100	100
Ramp time (min)	5	1	1	1
Hold time (min)	25	10	1	1
Power (%)^a^	60	20	10	0

^a^ – 100% power corresponds to 1450 W.

### Bland and Altman method

In the validation study of the method based on HR-CS FAAS for the determination of elements over a wide concentration range, sometimes up to 3 orders of magnitude (Cu, Pb, Zn), the comparison was made with ICP-OES as reference method using the Bland and Altman approach. It was initially applied in the medical field [56,57] and further used for comparative studies in other domains [58-62]. Unlike the linear regression, the Bland and Altman test can be applied for statistical evaluation of experimental data even when they are not uniformly distributed over a concentration range but grouped in several sub-domains, which may be the case of soil samples.

Setting up a Bland and Altman method to compare two data sets implies the following computing: (i) the average of repeated measurements for each sample and each method, and the within-sample standard deviation in each method (s_i HR-CS FAAS_ and s_i ICP-OES_); (ii) the within-method standard deviation

(1) $$s_{\text{wHR}-\text{CSFAAS}}=\sqrt{\frac{\sum s_{\text{iHR}-\text{CSFAAS}}^{2}}{n}}$$

(2) $$s_{\text{wICP}-\text{OES}}=\sqrt{\frac{\sum s_{\text{iICP}-\text{OES}}^{2}}{n}}$$

where n is the sample size; (iii) the differences between averages of measurements of each sample (Δ_i_) performed by the two methods, the bias estimated by the average difference ($\bar{\Delta }$) and its standard deviation (s_Δ_); (iv) the between-method standard deviation (s_B_) for (m) repeated measurements for each sample in each method

(3) $$s_{B}=\sqrt{s_{\Delta }^{2}+\left(1-\frac{1}{m}\right)s_{\text{wHR}-\text{CSFAAS}}^{2}+\left(1-\frac{1}{m}\right)s_{\text{wICP}-\text{OES}}^{2}}$$

(v) the limits of agreement of the results $\bar{\Delta }\pm 1.96s_{B}$ and the 95% CI of $\bar{\Delta }$ ($\bar{\Delta }\pm t\frac{s_{B}}{\sqrt{n}}$) where n is the sample size, while (t) the Student parameter for (n-1) freedom degrees; (vi) the 95% CI of limits of agreement of the mean difference ($\bar{\Delta }\pm 1.96s_{B}\pm t\cdot s_{\text{LL},\text{UL}}$) where s_LL, UL_ is the standard deviation of the lower and upper limits of agreement;

(4) $$s_{\text{LL},\text{UL}}=\sqrt{\frac{s_{B}^{2}}{n}+\frac{1.96^{2}}{{2s}_{B^{2}}}\left(\frac{s_{\Delta }^{4}}{n-1}+\frac{\left(m-1\right)s_{\text{wHR}-\text{CSFAAS}}^{4}}{nm^{2}}+\frac{\left(m-1\right)s_{\text{wICP}-\text{OES}}^{4}}{nm^{2}}\right)}$$

Finally the differences between methods are plotted against the averages of the two methods and the lines corresponding to bias, limits of agreement and their 95% CI respectively, are plotted.

According to the Bland and Altman test, there is no significant bias between methods when the differences of the results fall between the limits of agreement and the CI of the mean difference contains the zero value. Moreover, the 95% CI of the upper and lower limits should be reasonably narrow, which is very likely for a large sample size.

## Abbreviations

CCD: Charge coupled device; CRM: Certified reference material; HR-CS AAS: High-resolution continuum source atomic absorption spectrometry; HR-CS FAAS: High-resolution continuum source flame atomic absorption spectrometry; HR-CS GFAAS: High-resolution continuum source graphite furnace atomic absorption spectrometry; ICP-OES: Inductively coupled plasma optical emission spectrometry; LOD: Limit of detection; LOQ: Limit of quantification; LR-LS AAS: Low-resolution line source atomic absorption spectrometry; LSBC: Least-squares background correction; OES: Optical emission spectrometry; PTFE: Polytetrafluoroethylene; SNR: Signal-to-noise ratio.

## Competing interests

The authors declare that they have no competing interests.

## Authors’ contributions

TF – designed the study and coordinated the preparation of the manuscript, carried out the analysis by inductively coupled plasma optical emission spectrometry, co-worked on the analysis by high-resolution continuum source flame atomic absorption spectrometry and interpreted the results related to analytical performance; MP – performed the data comparison using the Bland and Altman method; worked on the sample preparation and analysis by optical emission and absorption spectrometry; RH – carried out the computational experiments; co-worked on the sample collection and preparation and helped to draft the manuscript. All authors read and approved the final manuscript.

## Authors’ information

TF is associate professor of instrumental analysis at the University Babes-Bolyai Cluj-Napoca, Faculty of Chemistry and Chemical Engineering, Romania. His research field includes the development of analytical methods by optical emission spectrometry in inductively coupled or capacitively coupled plasma sources for determining the priority hazardous components in environmental samples and materials. He has also interests in the development of miniaturized analytical instrumentation based on plasma microtorches for on-site analysis.

MP is associate professor of instrumental analysis at the University Babes-Bolyai Cluj-Napoca, Faculty of Chemistry and Chemical Engineering, Romania. Her area of interests covers the development of analytical methods by atomic spectrometry, toxicological analysis, and quality control and quality assurance in chemical analysis.

RH is a PhD student at the Babes-Bolyai University Cluj-Napoca, Romania and officer at the Department of chemicals and hazardous waste by the Regional Environmental Protection Agency, Cluj-Napoca. Her research interests cover the waste management and related monitoring approaches.

## Supplementary Material

Additional file 1 **Analytical results (mg/kg) for soil samples analysis by HR-CS FAAS and ICP-OES**^**a**^**.** Click here for file

Additional file 2 Bland and Altman plots. Click here for file
