# Supplementary material for: Validation of an analytical method based on the high-resolution continuum source flame atomic absorption spectrometry for the fast-sequential determination of several hazardous/priority hazardous metals in soil
Source: Chem Cent J. 2013 Mar 1;7:43. doi: 10.1186/1752-153X-7-43 (PMC3771560; doi:10.1186/1752-153X-7-43)
Supplement: Additional file 1 — Analytical results (mg/kg) for soil samples analysis by HR-CS FAAS and ICP-OES a . [file 1752-153X-7-43-S1.pdf]

Additional file 1 – Analytical results (mg/kg) for soil samples analysis by HR-CS FAAS and ICP-OES[a]

	Ag		Cd		Co		Cr		Cu		Ni		Pb		Zn	
	ICP-OES	HR-CS FAAS	ICP-OES	HR-CS FAAS	ICP-OES	HR-CS FAAS	ICP-OES	HR-CS FAAS	ICP-OES	HR-CS FAAS	ICP-OES	HR-CS FAAS	ICP-OES	HR-CS FAAS	ICP-OES	HR-CS FAAS
1	19.9±0.4	17.9±0.1	7.1±0.2	8.2±0.2	3.70±0.5	5.50±0.2	41.6±0.1	40.8±0.3	45.6±3.0	43.5±1.5	8.1±1.3	8.3±0.7	200±2	217±4	143±2	146±2
2	14.3±0.7	17.9±0.3	17.7±0.2	16.2±0.2	23.5±0.5	22.7±1.3	37.1±0.8	38.6±0.3	45.3±3.4	42.1±1.2	10.3±0.6	10.4±0.4	226±2	209±2	163±1	164±3
3	17.7±0.2	20.1±0.4	17.7±1.0	17.6±1.9	19.4±0.6	18.8±0.2	11.3±0.2	8.4±0.3	49.2±3.8	45.8±2.4	8.6±0.6	9.2±0.4	222±1	216±3	174±1	172±3
4	20.4±0.4	22.6±0.3	37.5±0.1	38.6±0.2	7.7±0.3	9.4±0.3	50.4±1.2	45.7±0.6	39.0±1.3	38.3±0.5	9.2±1.1	8.8±0.4	317±4	293±3	138±1	135±1
5	29.8±0.4	25.3±0.3	18.0±0.5	17.0±0.2	13.0±0.6	12.1±1.6	54.5±1.9	48.3±3.6	54.9±4.0	52.5±6.0	9.7±0.3	10.1±0.8	158±2	127±2	211±1	213±7
6	10.7±0.4	12.5±0.2	16.3±0.6	16.8±1.4	10.5±0.2	11.2±0.2	43.7±0.1	41.5±1.5	50.8±3.7	50.3±4.0	13.5±0.4	12.9±0.4	145±3	140±2	179±2	178±1
7	8.2±0.4	5.7±0.2	7.9±0.8	6.5±1.1	41.4±0.4	43.5±1.4	34.9±0.9	32.3±0.6	116±9	116±3	18.5±0.1	19.7±0.3	211±2	201±2	300±3	312±1
8	10.7±0.6	10.5±0.2	49.5±0.9	47.9±0.5	34.9±0.4	33.0±0.9	32.9±0.5	32.1±0.9	105±5	108±2	19.0±0.1	19.5±0.4	221±4	226±3	268±1	278±3
9	23.0±0.3	21.6±0.2	55.9±1.7	57.8±1.0	35.7±0.4	34.2±0.2	18.3±0.4	18.6±0.7	69.2±1.4	76.6±0.6	35.3±0.6	38.0±0.3	327±9	311±4	489±2	487±3
10	5.8±0.6	8.0±0.2	50.0±1.1	49.5±0.5	13.1±0.3	13.2±0.7	64.1±2.2	61.8±0.8	93.8±4.1	88.0±1.8	32.9±0.7	31.7±0.5	242±3	237±1	257±2	256±2
11	3.1±0.2	4.9±0.1	60.5±1.5	56.5±1.8	18.1±0.9	18.0±0.4	68.5±2.7	61.9±2.0	201±11	193±5	35.8±0.4	36.6±0.2	338±5	320±6	279±2	288±2
12	8.2±0.3	7.9±0.5	25.4±0.3	24.2±0.1	17.2±0.4	17.9±0.2	37.2±2.1	41.0±0.9	192±11	188±4	56.2±0.5	56.7±0.3	465±4	454±8	387±11	411±4
13	5.1±0.1	6.7±0.2	18.4±0.4	16.3±0.3	20.4±1.0	18.8±0.4	13.5±0.2	14.1±0.1	360±8	346±4	104±2	103±1	444±4	440±3	629±4	629±4
14	8.1±0.3	9.3±0.1	19.1±0.7	21.1±0.9	19.7±0.5	17.8±0.4	67.0±0.8	72.8±0.7	391±19	364±25	75.7±1.6	72.0±0.8	525±7	516±4	918±6	922±5

15	4.4±0.4	6.4±0.2	54.7±1.1	55.5±0.5	15.4±0.4	15.4±0.4	69.8±1.0	70.1±1.9	489±55	464±25	86.7±0.8	85.0±0.3	990±41	994±30	819±18	820±7
16	9.6±0.5	9.4±0.2	52.7±0.9	54.7±0.3	6.4±0.1	8.2±0.1	71.6±0.5	63.7±0.6	510±56	469±23	104±2	106±2	845±22	843±10	789±14	782±6
17	17.7±0.5	17.0±0.2	63.7±0.7	66.8±0.8	11.3±0.4	10.0±0.2	29.0±0.2	25.6±0.1	360±23	334±30	126±1	130±1	973±41	986±20	940±5	961±6
18	6.0±0.4	6.7±0.2	63.7±1.0	64.5±0.3	3.2±0.2	2.3±0.3	14.3±0.1	14.8±0.3	626±48	606±50	17.9±0.1	15.1±0.3	789±4	803±11	492±20	506±10
19	4.2±0.1	4.7±0.2	44.3±0.6	47.1±0.4	39.8±1.7	40.1±1.0	115±2	118.0±0.9	606±59	585±25	21.6±1.0	22.8±0.4	985±126	898±100	690±7	720±10
20	17.0±0.4	18.4±0.2	50.6±2.2	58.9±0.8	36.5±2.5	36.6±1.0	130±2	127.0±0.8	387±26	355±29	25.9±0.7	24.3±0.9	759±9	752±3	215±5	213±3
21	6.5±0.2	7.0±0.1	67.9±0.9	68.5±0.3	6.30±0.1	4.8±0.4	139±3	140.0±4.1	295±20	320±15	43.0±0.4	47.3±0.3	923±22	975±9	616±10	591±9
22	6.8±0.1	7.4±0.2	75.2±1.9	78.9±0.6	3.80±0.1	3.5±0.2	11.3±0.1	16.0±1.6	360±32	390±22	24.7±0.4	25.4±0.5	910±33	903±49	570±14	586±11
23	3.1±0.2	5.0±0.1	9.1±0.2	11.6±0.2	8.80±0.1	8.2±0.4	16.1±0.3	11.8±0.2	482±43	496±31	13.6±0.1	13.4±0.1	743±8	816±2	136±1	140±1
24	12.4±0.2	12.6±0.3	12.0±0.2	9.4±0.2	4.30±0.1	3.9±0.2	64.7±1.7	66.6±1.6	585±40	553±32	15.6±0.1	13.4±0.1	798±6	754±4	355±9	374±3
25	25.6±0.2	25.4±0.3	95.9±1.0	94.5±1.2	6.30±0.3	5.7±0.3	124.0±0.9	127.0±0.3	552±52	560±24	12.2±0.7	11.4±0.4	135±11	143±6	83.5±1.5	87.6±0.5
26									501±39	502±29			960±51	995±20	214±4	214±4
27									401±20	412±10			108±3	90±1	180±4	157±4
28									245±32	248±11			368±4	330±5	172±4	177±5
29									153±9	148±3			4309±150	4292±52	146±3	150±5
30									741±24	745±16			3890±160	4049±65	167±5	164±6
31									98.8±5.8	85.6±4.2			5717±190	5590±73	1093±15	1082±5
32									408±28	393±18			4719±507	4408±84	1492±20	1462±8

33									778±28	787±10			4249±135	4319±75	1458±15	1433±13
34									660±64	684±4			5830±107	6095±86	1372±13	1335±17
35									1464±17	1480±10			1621±32	1689±12	1594±8	1607±21
36									1319±25	1339±30			1691±221	1728±76	2197±15	2121±23
37									1608±58	1648±25			1496±27	1777±11	8005±200	8075±120
38									7946±102	7880±124			1799±10	1770±4	10627±150	10424±207
39									8328±148	8212±185			2044±47	2099±68	5052±188	5032±135
40									3421±85	3433±28			1842±21	1949±14	1870±53	1909±30
41									15783±335	15404±469			1125±126	1038±100	9544±250	9789±100
42									14605±452	14535±229			1050±51	1085±20	9100±240	8830±153
43									11882±194	11932±72			40772±1000	39191±400	3822±55	3785±19
44									13209±301	13636±164			35100±340	34471±175	6285±222	6196±166
45									12607±201	12368±250			13186±520	14210±185	1374±18	1396±21
46									4172±174	4101±116			48633±487	46651±187	1034±22	1038±6
47									6584±26	6552±46			43905±250	44035±132	1061±17	1073±10
48									21078±495	21688±452			31189±645	31338±407	7860±120	8069±140
49									21583±509	22012±554			28400±522	30449±913	8003±70	7885±24
50									12451±115	12731±153			56311±865	55408±443	1870±53	1909±15

51													55744±1032	57423±804		
52													28406±1089	30482±732		
53													49826±1079	50741±711		
54													12549±183	12539±76		
55													12430±130	12670±76		
