# Supplementary material for: Validation of an analytical method based on the high-resolution continuum source flame atomic absorption spectrometry for the fast-sequential determination of several hazardous/priority hazardous metals in soil
Source: Chem Cent J. 2013 Mar 1;7:43. doi: 10.1186/1752-153X-7-43 (PMC3771560; doi:10.1186/1752-153X-7-43)
Supplement: Additional file 2 — Bland and Altman plots. [file 1752-153X-7-43-S2.pdf]

Additional file 2 – Bland and Altman plots

(e) Cu (n=34 < 1000 mg/kg); (f) Cu (n=16 > 1000 mg/kg); (g) Ni (n=25); (h) Pb (n=28 < 1000 mg/kg); (i) Pb (n=12 between 1000-10000 mg/kg); (j) Pb (n=13 > 10000 mg/kg); (k) Zn (n=30 < 1000 mg/kg); (l) Zn (n=20 > 1000 mg/kg)

n– sample size.

The graph displays a scatter diagram of the differences plotted against the averages of the measurements in the two methods. Horizontal lines are drawn at the mean difference, and at the limits of agreement, which are defined as the mean difference ± 95% confidence interval.

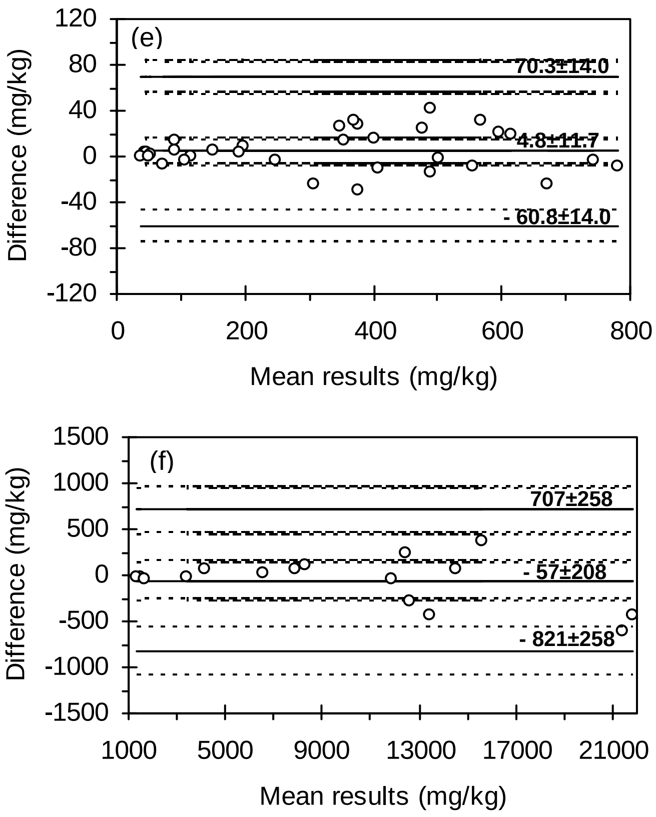

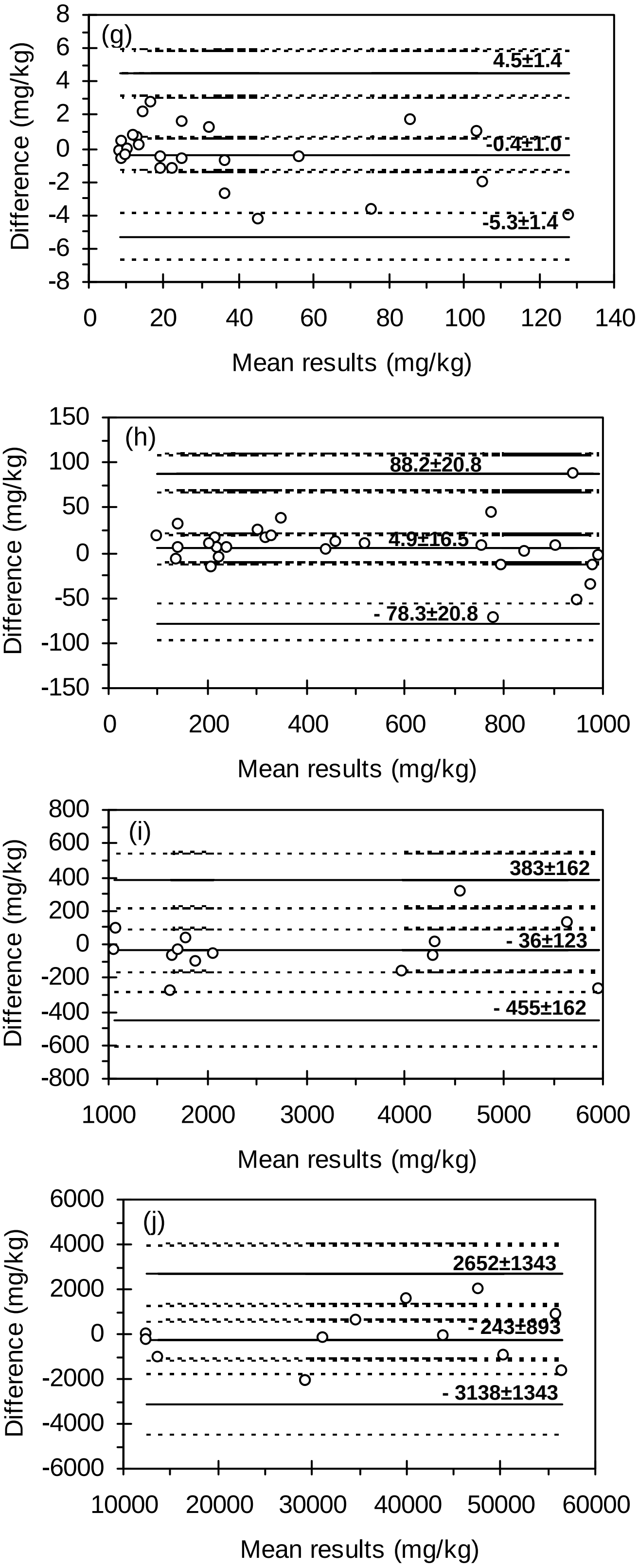
(g)
4.5±1.4
-0.4±1.0
-5.3±1.4
Difference (mg/kg)
Mean results (mg/kg)
(h)
88.2±20.8
4.9±16.5
- 78.3±20.8
Difference (mg/kg)
Mean results (mg/kg)
(i)
383±162
- 36±123
- 455±162
Difference (mg/kg)
Mean results (mg/kg)
(j)
2652±1343
- 243±893
- 3138±1343
Difference (mg/kg)
Mean results (mg/kg)

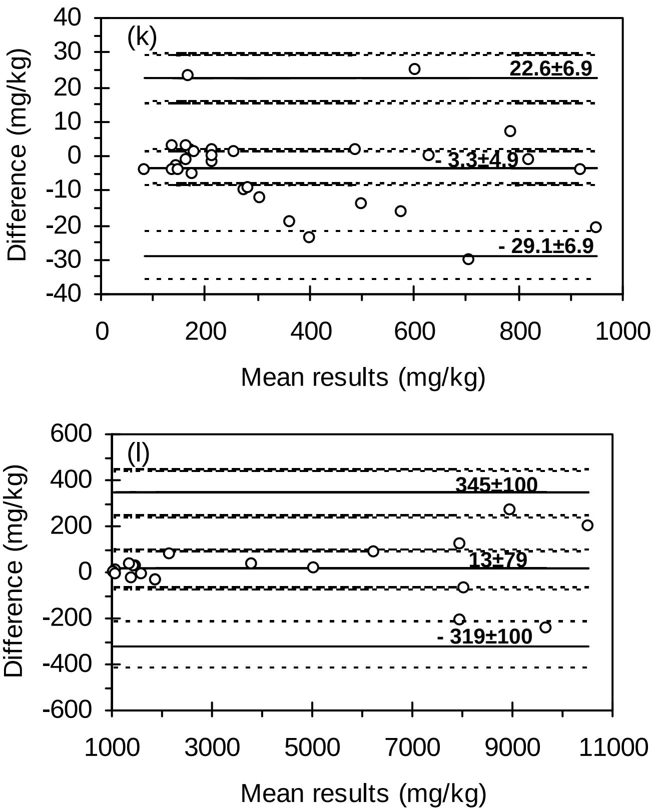
(k)
22.6±6.9
- 3.3±4.9
- 29.1±6.9
Difference (mg/kg)
Mean results (mg/kg)
(l)
345±100
13±79
- 319±100
Difference (mg/kg)
Mean results (mg/kg)
